# Supplementary figures and images for: Prism adaptation treatment to address spatial neglect in an intensive rehabilitation program: A randomized pilot and feasibility trial
Source: PLoS One. 2021 Jan 22;16(1):e0245425. doi: 10.1371/journal.pone.0245425 (PMC7822563; doi:10.1371/journal.pone.0245425)

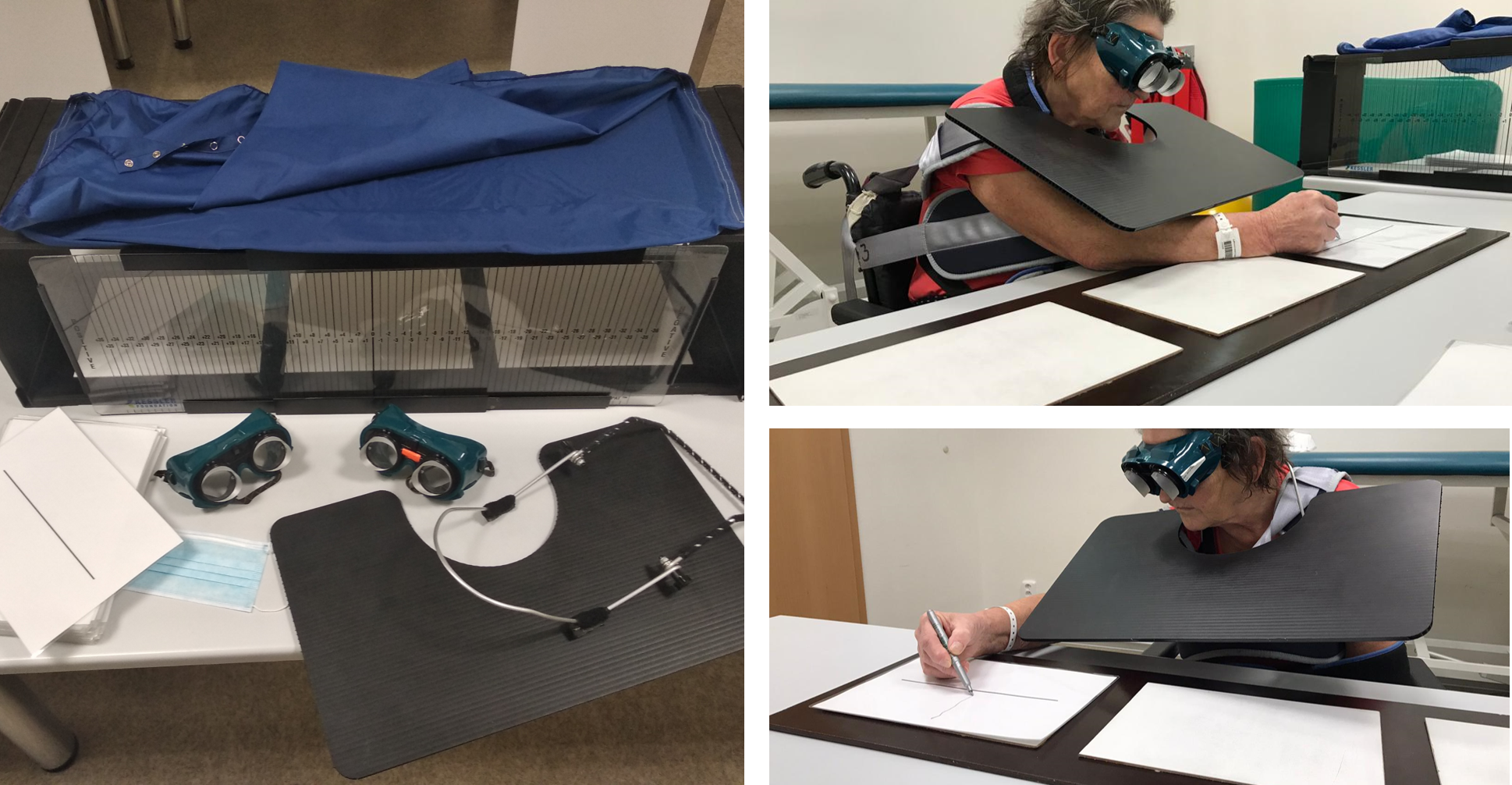

Supplement: S1 Fig — (TIF) [file pone.0245425.s001.tif]

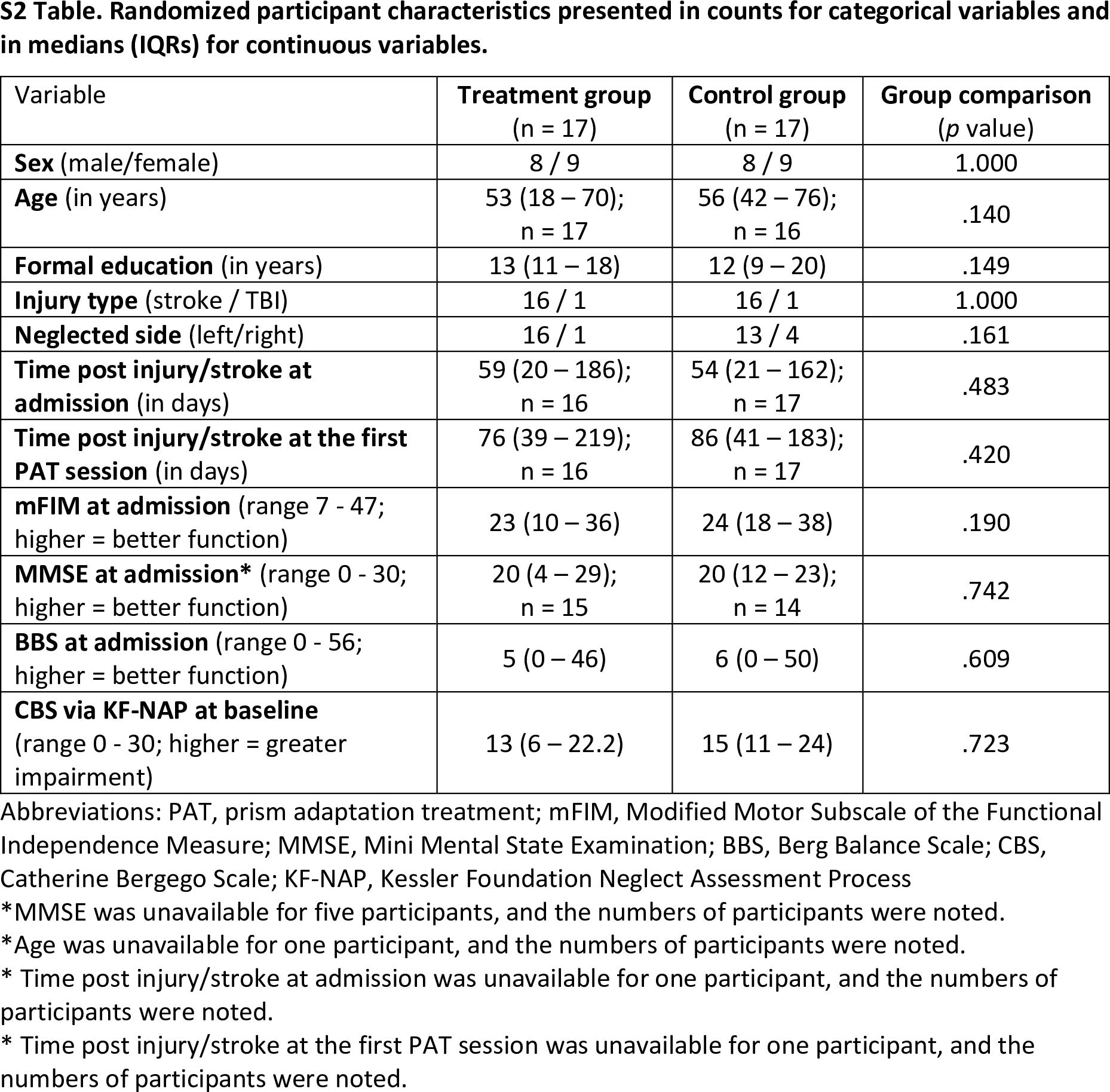

Supplement: S1 Table — (TIF) [file pone.0245425.s002.tif]
